# Supplementary material for: A Likelihood Approach for Real-Time Calibration of Stochastic Compartmental Epidemic Models
Source: PLoS Comput Biol. 2017 Jan 17;13(1):e1005257. doi: 10.1371/journal.pcbi.1005257 (PMC5240920; doi:10.1371/journal.pcbi.1005257)
Supplement: S1 File — (TAR.GZ) [file pcbi.1005257.s014.tar.gz › HSPH_Online-SI-Revision/MSS10/n1-extreme/n1-extreme_NEW2_table-4.pdf]

1	newly 3 weeks spec	116.	98.7	{47., 172.}	35.9828	0.195823	newly 3 weeks cumul	225.	204.22	{102., 319.}	36.4978	0.182814	newly 5 weeks spec	137.	113.9	{80., 138.}	19.708	0.102262	newly 5 weeks cumul	508.	437.05	{281., 602.}	23.8091	0.123994	newly 64 weeks cumul	896.	833.6	{715., 912.}	7.73438	0.0374274
2	newly 3 weeks spec	95.	99.56	{83., 114.}	11.2632	0.0475495	newly 3 weeks cumul	271.	288.	{251., 318.}	9.80074	0.0409286	newly 5 weeks spec	80.	76.48	{64., 91.}	11.6	0.0535387	newly 5 weeks cumul	464.	453.73	{413., 489.}	5.2694	0.0238017	newly 60 weeks cumul	752.	705.99	{653., 762.}	6.80452	0.0319249
3	newly 3 weeks spec	74.	66.8	{35., 94.}	26.5946	0.140861	newly 3 weeks cumul	153.	160.25	{98., 223.}	27.4837	0.12428	newly 5 weeks spec	91.	75.61	{48., 104.}	23.3516	0.12584	newly 5 weeks cumul	361.	311.1	{186., 424.}	22.5097	0.120767	newly 62 weeks cumul	847.	709.21	{558., 852.}	16.7214	0.0860264
4	newly 3 weeks spec	66.	62.91	{32., 83.}	24.4091	0.118308	newly 3 weeks cumul	162.	153.77	{88., 198.}	21.6728	0.103777	newly 5 weeks spec	58.	73.87	{38., 98.}	40.0862	0.157675	newly 5 weeks cumul	300.	298.61	{181., 378.}	19.6767	0.093059	newly 61 weeks cumul	732.	699.36	{437., 840.}	17.3361	0.0919747
5	newly 3 weeks spec	89.	71.07	{46., 96.}	24.2135	0.145183	newly 3 weeks cumul	241.	220.65	{162., 292.}	18.8589	0.0981589	newly 5 weeks spec	98.	51.13	{37., 68.}	47.8265	0.349864	newly 5 weeks cumul	447.	333.94	{251., 415.}	25.3602	0.145814	newly 54 weeks cumul	764.	466.55	{388., 547.}	38.9332	0.233092
6	newly 3 weeks spec	46.	22.25	{0., 47.}	56.3696	0.75999	newly 3 weeks cumul	105.	91.22	{21., 164.}	44.6476	0.289611	newly 5 weeks spec	44.	11.16	{0., 29.}	78.0909	1.33571	newly 5 weeks cumul	246.	117.6	{21., 215.}	53.878	0.4664	newly 59 weeks cumul	557.	137.63	{21., 249.}	75.4704	0.769872
7	newly 3 weeks spec	75.	81.47	{31., 119.}	38.2267	0.194508	newly 3 weeks cumul	185.	236.95	{129., 316.}	43.3027	0.180467	newly 5 weeks spec	81.	57.96	{10., 94.}	37.358	0.336409	newly 5 weeks cumul	355.	366.39	{188., 494.}	29.5803	0.149896	newly 63 weeks cumul	753.	550.65	{230., 816.}	30.8831	0.211219
8	newly 3 weeks spec	76.	53.69	{38., 73.}	29.8026	0.16614	newly 3 weeks cumul	224.	155.97	{120., 195.}	30.6116	0.165906	newly 5 weeks spec	72.	49.04	{36., 60.}	32.0556	0.176885	newly 5 weeks cumul	382.	257.17	{204., 322.}	32.678	0.178466	newly 57 weeks cumul	642.	509.14	{434., 569.}	20.6947	0.103319
9	newly 3 weeks spec	86.	35.71	{0., 80.}	62.2674	0.789554	newly 3 weeks cumul	199.	114.14	{18., 222.}	52.2714	0.450262	newly 5 weeks spec	96.	30.54	{0., 78.}	70.5625	1.22447	newly 5 weeks cumul	400.	176.77	{18., 359.}	57.3925	0.556898	newly 64 weeks cumul	731.	287.01	{18., 698.}	62.4227	0.674887
10	newly 3 weeks spec	73.	63.5	{48., 78.}	16.0822	0.0793084	newly 3 weeks cumul	217.	172.69	{143., 206.}	20.5945	0.104267	newly 5 weeks spec	62.	63.41	{52., 76.}	12.7903	0.0547147	newly 5 weeks cumul	362.	301.46	{258., 343.}	16.989	0.0837779	newly 57 weeks cumul	751.	654.43	{604., 691.}	12.8589	0.0604963
11	newly 3 weeks spec	88.	104.13	{70., 134.}	33.1023	0.185227	newly 3 weeks cumul	241.	271.28	{185., 346.}	26.3071	0.134692	newly 5 weeks spec	81.	83.66	{32., 109.}	30.8395	0.208111	newly 5 weeks cumul	416.	454.96	{262., 554.}	23.524	0.132707	newly 63 weeks cumul	680.	695.37	{370., 851.}	24.6515	0.151638
12	newly 3 weeks spec	65.	59.83	{27., 98.}	34.6923	0.185471	newly 3 weeks cumul	159.	122.51	{61., 188.}	31.8805	0.194112	newly 5 weeks spec	109.	92.87	{45., 128.}	24.4312	0.151735	newly 5 weeks cumul	387.	295.32	{163., 426.}	28.9302	0.181032	newly 64 weeks cumul	698.	818.96	{709., 903.}	19.5989	0.0787537
13	newly 3 weeks spec	49.	37.8	{24., 53.}	27.7143	0.151021	newly 3 weeks cumul	125.	100.84	{75., 137.}	24.736	0.12672	newly 5 weeks spec	41.	46.19	{29., 61.}	25.878	0.102932	newly 5 weeks cumul	222.	188.99	{140., 240.}	21.1036	0.104427	newly 57 weeks cumul	659.	602.37	{479., 669.}	9.38847	0.0466132
14	newly 3 weeks spec	113.	80.07	{63., 97.}	29.708	0.158486	newly 3 weeks cumul	293.	218.39	{175., 268.}	25.8123	0.134108	newly 5 weeks spec	92.	71.84	{57., 87.}	22.413	0.115043	newly 5 weeks cumul	501.	369.63	{320., 437.}	26.2216	0.13493	newly 61 weeks cumul	659.	668.11	{605., 744.}	6.81184	0.0295382
15	newly 3 weeks spec	61.	36.69	{18., 56.}	42.7049	0.282703	newly 3 weeks cumul	196.	104.85	{61., 152.}	46.7398	0.306499	newly 5 weeks spec	65.	35.92	{14., 56.}	45.4462	0.320847	newly 5 weeks cumul	338.	178.36	{94., 259.}	47.2308	0.313338	newly 56 weeks cumul	615.	411.26	{276., 585.}	34.7317	0.197987
16	newly 3 weeks spec	79.	110.61	{82., 137.}	46.519	0.190296	newly 3 weeks cumul	198.	275.62	{204., 337.}	43.8384	0.167136	newly 5 weeks spec	51.	98.69	{79., 119.}	98.7647	0.328587	newly 5 weeks cumul	335.	484.41	{411., 553.}	49.0299	0.187567	newly 63 weeks cumul	677.	770.01	{658., 860.}	20.4328	0.102501
17	newly 3 weeks spec	73.	57.93	{35., 79.}	25.5205	0.140218	newly 3 weeks cumul	179.	142.02	{100., 181.}	24.3799	0.127942	newly 5 weeks spec	61.	70.39	{43., 91.}	28.5082	0.133689	newly 5 weeks cumul	321.	276.85	{199., 340.}	18.3271	0.0944409	newly 60 weeks cumul	748.	697.89	{591., 790.}	10.5789	0.0542951
18	newly 3 weeks spec	100.	77.37	{43., 110.}	30.91	0.204033	newly 3 weeks cumul	211.	199.4	{129., 286.}	25.3555	0.122864	newly 5 weeks spec	103.	76.87	{20., 108.}	28.068	0.298409	newly 5 weeks cumul	426.	356.88	{233., 479.}	22.2207	0.127664	newly 63 weeks cumul	855.	660.27	{297., 832.}	23.0211	0.156276
19	newly 3 weeks spec	67.	69.91	{23., 108.}	40.9701	0.277383	newly 3 weeks cumul	165.	182.62	{75., 284.}	38.9212	0.188482	newly 5 weeks spec	77.	59.96	{9., 103.}	40.8571	0.372403	newly 5 weeks cumul	334.	311.48	{117., 457.}	31.006	0.186508	newly 64 weeks cumul	820.	506.43	{212., 852.}	40.0232	0.302227
20	newly 3 weeks spec	98.	72.71	{58., 91.}	26.2347	0.140303	newly 3 weeks cumul	274.	222.19	{185., 270.}	19.4562	0.0981608	newly 5 weeks spec	84.	57.96	{49., 71.}	31.	0.17357	newly 5 weeks cumul	458.	345.05	{307., 400.}	24.6616	0.127732	newly 57 weeks cumul	701.	579.28	{539., 628.}	17.3638	0.0896516
21	newly 3 weeks spec	44.	36.09	{13., 55.}	34.2045	0.255413	newly 3 weeks cumul	115.	95.09	{46., 137.}	31.3826	0.178633	newly 5 weeks spec	43.	39.75	{8., 63.}	34.9535	0.293948	newly 5 weeks cumul	219.	173.99	{79., 244.}	29.3927	0.183726	newly 60 weeks cumul	654.	519.16	{171., 729.}	26.4709	0.197414
22	newly 3 weeks spec	79.	95.45	{70., 119.}	25.7342	0.0992455	newly 3 weeks cumul	241.	274.87	{219., 330.}	20.0871	0.080711	newly 5 weeks spec	63.	73.3	{57., 89.}	22.127	0.0877908	newly 5 weeks cumul	388.	434.24	{349., 503.}	17.1289	0.0705123	newly 61 weeks cumul	683.	653.21	{538., 751.}	10.4729	0.0491946
23	newly 3 weeks spec	72.	86.04	{58., 110.}	27.6667	0.106309	newly 3 weeks cumul	168.	205.63	{148., 251.}	28.1131	0.105871	newly 5 weeks spec	79.	92.31	{71., 113.}	24.2152	0.0972144	newly 5 weeks cumul	346.	393.13	{303., 482.}	20.0838	0.0805676	newly 63 weeks cumul	679.	755.16	{653., 863.}	17.1664	0.0724479
24	newly 3 weeks spec	106.	118.85	{83., 159.}	26.7642	0.112626	newly 3 weeks cumul	236.	265.89	{176., 351.}	27.3008	0.112946	newly 5 weeks spec	137.	110.97	{80., 131.}	19.9635	0.127235	newly 5 weeks cumul	531.	499.99	{390., 611.}	14.3032	0.0705444	newly 64 weeks cumul	842.	801.63	{654., 882.}	7.82542	0.0416128
25	newly 3 weeks spec	63.	66.94	{45., 86.}	21.1429	0.09128	newly 3 weeks cumul	139.	160.27	{116., 203.}	24.0216	0.0951657	newly 5 weeks spec	68.	80.28	{59., 98.}	24.2059	0.094173	newly 5 weeks cumul	276.	315.81	{227., 375.}	21.721	0.0874837	newly 61 weeks cumul	696.	727.96	{634., 805.}	8.69253	0.0365925
26	newly 3 weeks spec	131.	80.41	{52., 101.}	38.771	0.227004	newly 3 weeks cumul	280.	172.23	{128., 215.}	38.8607	0.224439	newly 5 weeks spec	153.	103.06	{79., 121.}	32.6405	0.178031	newly 5 weeks cumul	589.	373.83	{283., 446.}	36.5314	0.20464	newly 63 weeks cumul	887.	866.8	{793., 908.}	3.83315	0.0173725
27	newly 3 weeks spec	61.	43.18	{8., 71.}	36.1639	0.39852	newly 3 weeks cumul	160.	136.68	{35., 200.}	30.95	0.209388	newly 5 weeks spec	54.	36.72	{0., 65.}	44.2222	0.560156	newly 5 weeks cumul	277.	213.1	{42., 324.}	32.7509	0.249396	newly 58 weeks cumul	659.	377.37	{42., 618.}	42.7481	0.390152
28	newly 3 weeks spec	77.	64.65	{29., 99.}	31.2338	0.209922	newly 3 weeks cumul	250.	184.19	{95., 267.}	29.62	0.183504	newly 5 weeks spec	99.	51.81	{16., 87.}	47.8283	0.450001	newly 5 weeks cumul	460.	296.16	{160., 438.}	36.0957	0.238916	newly 62 weeks cumul	803.	470.94	{208., 729.}	41.3973	0.300323
29	newly 3 weeks spec	145.	57.89	{45., 73.}	60.0759	0.407119	newly 3 weeks cumul	304.	140.34	{116., 170.}	53.8355	0.34079	newly 5 weeks spec	113.	72.63	{54., 87.}	35.7257	0.199426	newly 5 weeks cumul	568.	280.95	{224., 335.}	50.537	0.310438	newly 59 weeks cumul	875.	786.39	{758., 816.}	10.1269	0.0466217
30	newly 3 weeks spec	52.	36.12	{20., 48.}	32.0385	0.202235	newly 3 weeks cumul	145.	133.83	{97., 168.}	16.7241	0.0843949	newly 5 weeks spec	41.	22.39	{11., 33.}	45.878	0.342525	newly 5 weeks cumul	245.	186.02	{125., 233.}	24.6694	0.136951	newly 54 weeks cumul	398.	252.95	{157., 315.}	36.4598	0.215991
31	newly 3 weeks spec	115.	76.86	{25., 113.}	34.8696	0.312758	newly 3 weeks cumul	258.	200.32	{98., 281.}	27.7209	0.194206	newly 5 weeks spec	94.	66.88	{4., 104.}	33.9149	0.430288	newly 5 weeks cumul	471.	341.41	{146., 467.}	28.7877	0.2202	newly 63 weeks cumul	796.	610.48	{227., 849.}	27.5553	0.240752
32	newly 3 weeks spec	106.	66.77	{28., 100.}	37.9717	0.294748	newly 3 weeks cumul	229.	173.21	{82., 242.}	27.524	0.18639	newly 5 weeks spec	128.	64.98	{19., 98.}	49.2344	0.444556	newly 5 weeks cumul	487.	306.95	{138., 429.}	36.9713	0.259198	newly 63 weeks cumul	773.	565.52	{285., 795.}	28.4864	0.214016
33	newly 3 weeks spec	60.	49.31	{30., 67.}	24.5833	0.136682	newly 3 weeks cumul	152.	123.02	{84., 163.}	23.0395	0.124692	newly 5 weeks spec	78.	61.51	{41., 80.}	23.3718	0.12653	newly 5 weeks cumul	313.	240.88	{168., 309.}	24.4026	0.132605	newly 59 weeks cumul	799.	716.12	{666., 779.}	10.5607	0.0493384
34	newly 3 weeks spec	85.	62.93	{20., 92.}	30.1529	0.265073	newly 3 weeks cumul	234.	170.32	{92., 228.}	30.453	0.19104	newly 5 weeks spec	96.	61.7	{8., 90.}	36.3333	0.415443	newly 5 weeks cumul	427.	297.89	{136., 389.}	31.1874	0.215124	newly 62 weeks cumul	764.	589.9	{173., 813.}	25.9817	0.214832
35	newly 3 weeks spec	73.	90.19	{48., 124.}	39.4384	0.189485	newly 3 weeks cumul	181.	237.21	{153., 311.}	40.6464	0.160599	newly 5 weeks spec	79.	71.37	{14., 104.}	33.9873	0.260725	newly 5 weeks cumul	361.	393.32	{230., 499.}	24.831	0.120882	newly 63 weeks cumul	749.	639.58	{276., 856.}	25.7597	0.162645
36	newly 3 weeks spec	81.	73.49	{49., 94.}	18.679	0.0956437	newly 3 weeks cumul	195.	192.8	{140., 237.}	16.1333	0.0743177	newly 5 weeks spec	83.	68.34	{47., 90.}	21.9036	0.115035	newly 5 weeks cumul	376.	335.2	{242., 401.}	14.9096	0.0752545	newly 60 weeks cumul	755.	623.92	{544., 725.}	17.7219	0.0876117
37	newly 3 weeks spec	78.	54.81	{14., 81.}	32.8077	0.273137	newly 3 weeks cumul	218.	176.12	{101., 236.}	25.8165	0.163881	newly 5 weeks spec	65.	42.5	{10., 67.}	36.8923	0.35954	newly 5 weeks cumul	380.	268.31	{132., 358.}	29.8395	0.2004	newly 55 weeks cumul	728.	435.6	{184., 649.}	40.1648	0.290696
38	newly 3 weeks spec	93.	89.22	{75., 106.}	11.7634	0.0537083	newly 3 weeks cumul	234.	236.89	{198., 281.}	11.2778	0.0484455	newly 5 weeks spec	114.	77.82	{64., 90.}	31.7368	0.169469	newly 5 weeks cumul	462.	401.65	{351., 459.}	14.0801	0.0675452	newly 60 weeks cumul	754.	723.3	{653., 778.}	5.45093	0.024983
39	newly 3 weeks spec	89.	78.84	{50., 106.}	21.9551	0.110533	newly 3 weeks cumul	179.	169.	{105., 224.}	20.3017	0.0973696	newly 5 weeks spec	117.	103.64	{81., 122.}	13.6581	0.0670254	newly 5 weeks cumul	414.	372.01	{260., 464.}	15.6691	0.0776471	newly 63 weeks cumul	855.	847.53	{748., 907.}	5.58713	0.025081
40	newly 3 weeks spec	70.	65.84	{20., 104.}	42.3429	0.301163	newly 3 weeks cumul	153.	228.79	{126., 317.}	63.5752	0.245305	newly 5 weeks spec	66.	40.18	{1., 74.}	46.1515	0.55264	newly 5 weeks cumul	299.	322.25	{153., 474.}	41.1806	0.221088	newly 61 weeks cumul	575.	436.08	{160., 700.}	39.2417	0.29222
41	newly 3 weeks spec	45.	34.02	{22., 46.}	27.6	0.15063	newly 3 weeks cumul	135.	93.98	{70., 124.}	30.4148	0.166461	newly 5 weeks spec	55.	33.03	{23., 44.}	40.0545	0.237377	newly 5 weeks cumul	248.	159.38	{119., 201.}	35.7339	0.200191	newly 56 weeks cumul	562.	502.22	{437., 556.}	11.1601	0.0542446
42	newly 3 weeks spec	88.	90.14	{62., 113.}	18.8636	0.0852844	newly 3 weeks cumul	235.	206.25	{143., 249.}	18.5234	0.0930051	newly 5 weeks spec	105.	97.91	{83., 116.}	11.381	0.0586331	newly 5 weeks cumul	462.	403.24	{304., 475.}	15.3853	0.0778169	newly 64 weeks cumul	862.	818.55	{780., 880.}	5.91067	0.0340555
43	newly 3 weeks spec	82.	86.85	{54., 122.}	25.061	0.104133	newly 3 weeks cumul	219.	187.79	{125., 257.}	22.8904	0.115474	newly 5 weeks spec	75.	104.68	{86., 121.}	41.0667	0.146969	newly 5 weeks cumul	390.	396.65	{289., 488.}	14.8282	0.0646401	newly 63 weeks cumul	860.	843.69	{746., 902.}	5.61047	0.025427
44	newly 3 weeks spec	86.	103.56	{85., 122.}	22.4884	0.0859725	newly 3 weeks cumul	240.	273.02	{226., 325.}	16.8417	0.0662761	newly 5 weeks spec	76.	88.74	{75., 102.}	18.2105	0.0708421	newly 5 weeks cumul	408.	460.24	{407., 520.}	14.2892	0.0569351	newly 61 weeks cumul	762.	783.04	{723., 827.}	5.67454	0.0243737
45	newly 3 weeks spec	79.	94.48	{62., 131.}	31.8481	0.121183	newly 3 weeks cumul	203.	207.54	{126., 288.}	25.6946	0.114393	newly 5 weeks spec	105.	106.73	{80., 130.}	14.2762	0.0653586	newly 5 weeks cumul	422.	424.04	{314., 530.}	17.6161	0.0800541	newly 64 weeks cumul	886.	798.66	{707., 892.}	10.2641	0.0518033
46	newly 3 weeks spec	93.	84.73	{34., 130.}	34.0323	0.229458	newly 3 weeks cumul	223.	229.79	{89., 367.}	35.3408	0.175017	newly 5 weeks spec	79.	60.35	{13., 99.}	36.0633	0.32882	newly 5 weeks cumul	409.	365.76	{151., 528.}	27.3056	0.161606	newly 61 weeks cumul	815.	531.87	{246., 814.}	35.2061	0.246175
47	newly 3 weeks spec	63.	67.39	{41., 95.}	31.2857	0.142936	newly 3 weeks cumul	164.	158.65	{97., 223.}	26.5305	0.129032	newly 5 weeks spec	67.	72.71	{37., 97.}	29.3582	0.132757	newly 5 weeks cumul	308.	303.57	{187., 410.}	23.8994	0.114963	newly 62 weeks cumul	785.	678.76	{467., 847.}	17.3019	0.100175
48	newly 3 weeks spec	60.	66.02	{51., 84.}	18.6667	0.0747255	newly 3 weeks cumul	141.	175.36	{142., 214.}	25.6312	0.0952304	newly 5 weeks spec	68.	69.83	{53., 84.}	15.6029	0.0692313	newly 5 weeks cumul	280.	315.81	{261., 377.}	16.5321	0.065503	newly 62 weeks cumul	672.	709.85	{633., 778.}	10.2277	0.0444424
49	newly 3 weeks spec	96.	39.4	{0., 87.}	60.8333	0.834151	newly 3 weeks cumul	261.	146.06	{15., 262.}	47.6858	0.426627	newly 5 weeks spec	115.	20.21	{0., 54.}	82.4261	1.53911	newly 5 weeks cumul	493.	196.06	{16., 375.}	60.2312	0.58346	newly 64 weeks cumul	853.	237.64	{16., 427.}	72.1407	0.764623
50	newly 3 weeks spec	57.	30.38	{2., 61.}	52.7368	0.565117	newly 3 weeks cumul	158.	125.91	{32., 205.}	35.7278	0.262963	newly 5 weeks spec	54.	13.28	{0., 31.}	76.4074	1.19358	newly 5 weeks cumul	272.	160.48	{34., 268.}	42.9559	0.355854	newly 62 weeks cumul	579.	178.91	{38., 296.}	69.1002	0.635252
